# Indirect role of climatic suitability in mediating the effects of plant characteristics on naturalization success of cultivated alien plants in Southern Africa

**DOI:** 10.1007/s10530-025-03677-3

**Published:** 2025-09-25

**Authors:** Sarah-Olivia Peter, Franz Essl, Bernd Lenzner, Mark van Kleunen, Ali Omer

**Affiliations:** 1 Division of BioInvasions, Global Change and Macroecology, Department of Botany and Biodiversity Research, University of Vienna, Rennweg 14, 1030 Vienna, Austria; 2 Department of Agronomy and Plant Breeding II, Organic Farming with Focus on Sustainable Soil Use, Justus-Liebig-Universität Gießen, Gießen, Deutschland; 3 Ecology, Department of Biology, University of Konstanz, Universitätsstraße 10, 78464 Konstanz, Germany; 4 Zhejiang Key laboratory for Restoration of Damaged Coastal Ecosystems & Zhejiang Provincial Key Laboratory of Plant Evolutionary Ecology and Conservation, Taizhou University, Taizhou, 318000 China; 5 Department of Forest Management, Faculty of Forestry, University of Khartoum, North Khartoum, 13314 Sudan

**Keywords:** Biological invasion, Non-native plants, Ornamental plants, Southern Africa, Species distribution modeling

## Abstract

The rapid expansion of global trade, tourism, and human mobility has increased the introduction of alien plants into new regions. Here, we assessed the role of plant characteristics and climatic suitability in the naturalization success of 1,407 cultivated alien plants in Southern Africa. We used mediation analysis with climate suitability as a mediator to quantify the direct and indirect effects of plant characteristics, including phylogenetic relatedness, seed mass, plant height, native origins, native range size, and growth forms on naturalization success. We found that naturalized species have higher climatic suitability compared to non-naturalized ones. Additionally, seed mass, plant height, short-lived herbaceous growth form, and native range size are positively associated with naturalization success. In contrast, phylogenetic relatedness and a native origin in Europe were negatively associated with naturalization success. These associations were indirectly mediated by climatic suitability, with indirect effects accounting for more than 30% of the total effect in all cases. Our study underscores the significance of considering the role of climatic suitability for a comprehensive understanding of how plant characteristics impact the naturalization success of alien plants.

**Supplementary Information:**

The online version contains supplementary material available at 10.1007/s10530-025-03677-3.

## Introduction

The accelerating pace of global trade, tourism, and human mobility has led to a substantial increase in the exchange of biota between historically isolated regions (Seebens et al. 2017), and hence the ubiquitous presence of alien plant species introduced by humans (Banks et al. 2015; Hulme 2021; Perrings et al. 2005). According to van Kleunen et al. (2015), over 13,000 of these introduced plant species have established self-sustaining populations outside their native ranges, i.e., they have become naturalized (Richardson et al. 2000). A subset of these naturalized species has become invasive and poses a significant threat to global biodiversity and ecosystem functioning (Roy et al., 2023). Therefore, investigating the drivers of plant naturalization success has become a major task for science and environmental management.

Environmental similarity between native and non-native ranges plays an important role in the naturalization success of alien species. For example, climatic suitability of the non-native range is positively associated with the naturalization success of Chinese woody species in multiple countries (Feng et al. 2016). Moreover, climatic suitability has also been identified as a key driver of naturalization success at the global scale (Essl et al. 2019). Similarly, climatic suitability is expected to increase for the currently cultivated alien plants in Europe under climate change, which might increase their naturalization risk (Dullinger et al. 2017). Environmental similarity, including climate matching between native and introduced ranges is, thus, an essential factor for the naturalization and spread of alien plants (Richardson and Pyšek 2012).

Several studies have investigated the associations between plant characteristics and naturalization success. For instance, naturalization success was shown to be negatively correlated with phylogenetic distance to the native flora, as alien plants closely related to native ones are more likely to be preadapted to the new environment (Divíšek et al. 2018; Li et al. 2015; Omer et al. 2022). Functional traits, such as seed mass, plant height, and specific leaf area, were also found to be associated with plant naturalization success (Mayer et al. 2017). Moreover, geographical characteristics, such as native origins and native range size, were also recognized as important drivers of naturalization success (Dehnen-Schmutz et al. 2007; Kinlock et al. 2022). Some of these species’s characteristics may affect naturalization success because they influence the ability of plants to successfully cope with the prevailing environmental conditions in the new range (Reich 2014). In other words, species with specific characteristics might be more likely to naturalize due to their increased likelihood of passing the environmental filters. However, how climatic suitability mediates the associations between species characteristics and naturalization success has not been tested explicitly.

The introduction of living organisms into new regions has occurred through various means and for different purposes (Hulme et al. 2008). However, one of the dominant pathways for vascular plants is intentional introduction for cultivation (Faulkner et al. 2020; Lambdon et al. 2008). These species are grown in public spaces and have economic value (van Kleunen et al. 2018, 2020), and plant checklists of cultivated alien plants offer unique opportunities for studying naturalization risks.

Here, we aimed to disentangle the direct effects of plant characteristics and the indirect effects mediated by climatic suitability on naturalization success of cultivated alien plants on a sub-continental scale. We used a checklist of 1,407 alien plants that are known to be cultivated in at least one of the 10 countries in the region of Southern Africa. To specify the direct and indirect drivers of naturalization, we hypothesized that naturalization success is associated with climatic suitability and plant characteristics, mainly geographical origin and native range size, functional traits (i.e., growth form, seed mass, height and specific leaf area) and phylogenetic relatedness to the native flora. Furthermore, we hypothesized that climatic suitability acts as a filter determining the prevalence of plant traits within the naturalized flora pool. Accordingly, we employed a mediation analysis framework to disentangle the indirect effects mediated through climatic suitability from the direct effects of plant characteristics on naturalization success.

## Methods

### Study area

This study focuses on the region of Southern Africa, which has an area of approximately 4,000,000 km^2^. This region includes ten countries: Angola, Botswana, Eswatini (former Swaziland), Lesotho, Malawi, Mozambique, Namibia, South Africa, Zambia and Zimbabwe. The largest and most diverse country, South Africa, is split into nine provinces because data on species occurrence in these nine provinces was available; thus, a total of 18 different regions for Southern Africa were used (Fig. S1). Southern Africa has a long history of plant introductions, with some introductions dating back to the arrival of the Dutch in 1652 (Henderson 2006). The region of Southern Africa contains several biodiversity hotspots (Hu et al. 2021), including a distinct floral kingdom (Capensis).

### Cultivated alien flora of Southern Africa

This study is based on a checklist of species that have been reported to be cultivated (i.e., primarily as ornamental plants) in at least one of the 10 countries in Southern Africa. We compiled this checklist from the book ‘Cultivated Plants of Southern Africa’ (Glen 2002)*.* To ensure the comparability of this species list with other data sources (see below), the taxonomic names in this list were harmonized using The Plant List (v.1.1 < http://www.theplantlist.org/ >) and the R package “Taxonstand" (Cayuela et al. 2019). Species native to Southern Africa were removed based on native range information extracted from the Global Inventory of Floras and Traits database (GIFT; Weigelt et al. 2020). Intraspecific taxa were rounded to their species level. Species with insufficient data to run the species distribution models were also removed, as were ferns and Gymnosperms. Therefore, this study’s final list of species contained 1,407 cultivated alien species.

Furthermore, we extracted the naturalization status for each of the cultivated alien species in the region of Southern Africa, that is, whether cultivated alien species have established and formed self-sustaining populations in at least one country in Southern Africa, from the Global Naturalized Alien Flora Database (GloNAF). GloNAF is the most comprehensive database of naturalized alien plants and contains information on over 13,000 naturalized vascular plant taxa in more than 1,000 regions globally. GloNAF is based on over 210 alien flora checklists of different geographic regions (e.g., countries, states, provinces, districts, islands) (van Kleunen et al. 2019).

### Plant characteristics data

Data on cultivated alien plant characteristics was collected or calculated for all introduced alien plant species (Table S1). For geographical origin and native range size, we compiled data from the Plants of the World Online database (POWO 2023; www.plantsoftheworldonline.org/). Regions of origin were assigned to continents according to the TDWG level 1 classification (Brummitt 2001). To estimate the native range size for the cultivated alien plants, we used the number of TDWG level-3 regions (*n* = 369; Brummitt 2001) to which a species is native. Growth form data was gathered from multiple sources (see Omer et al. 2021). We then assigned each species to one or more of the following seven categories: short-lived herb, long-lived herb, free-standing woody, aquatic, climber, epiphyte and parasite. For functional traits, specific leaf area, height and seed mass were chosen as they capture crucial variation in plant strategies (Díaz et al. 2016). The data on this was collected from the TRY database (Kattge et al. 2020) and supplemented with other sources to close gaps in data coverage (see Omer et al. 2021).

### Phylogenetic relatedness to the native flora

To assess the phylogenetic relatedness between alien and native plant species, we first constructed a phylogenetic tree of all native and alien cultivated angiosperms in Southern Africa with accepted names according to The Plant List. As a basis for this phylogeny, we used the currently most comprehensive time-calibrated phylogeny of seed plants (Smith and Brown 2018). Then, for each alien cultivated species, we calculated three commonly used phylogenetic distance indices that provide information on the phylogenetic composition at the tips and deeper branches of the tree (Thuiller et al. 2010). To include the structures at the deeper branches of the phylogenetic tree, the mean phylogenetic distance to the native flora (PD_mean_) was calculated. This measure assumes an equal contribution of the entire native flora to the success or failure of the alien species, regardless of the abundance of the natives. The second measure included is the mean weighted phylogenetic distance to the native flora (PD_wmean_). This measure assumes that the abundance of the native species plays a role, as more prevalent native species are more likely to interact with alien species. The phylogenetic distance is therefore weighted by the occurrence frequency of the native species. For this measure, the number of occurrences in the Southern African subregions (n = 18), according to GIFT, was used to calculate PD_wmean_. Finally, phylogenetic distance to the nearest native species (PD_min_) was calculated to show the phylogenetic composition at the tree tip. According to this measure, alien species success is determined by the distance to its closest relative, as they are most likely to share similar resource requirements, mutualists and enemies (Divíšek et al. 2018; Thuiller et al. 2010). We excluded PD_wmean_ from our further analysis as it is highly correlated with PD_min_ (Fig. S2).

### Climatic suitability assessment

We assessed the climatic suitability of Southern Africa for each of the 1,407 cultivated alien species using species distribution models (SDMs). In brief, we collected species occurrence data from the Global Biodiversity Information Facility (GBIF.org, accessed 2021, 10.15468/dl.9jsscb), six bioclimatic predictors from WorldClim version 2.1 (Fick et al. 2017) and human population density (Jones et al. 2020) as a proxy of propagule pressure. We then used the BIOMOD2 platform, which was implemented in the ‘biomod2’ R package version 3.4–6 (Thuiller et al. 2009), to combine bioclimatic variables and human population density with presence records and randomly generated pseudo-absences. As a result, climatic suitability was derived as the number of grid cells projected to be suitable for each cultivated alien species. See Supporting Information for additional details regarding SDMs and climatic suitability assessment.

### Statistical analysis

Our primary objective was to investigate whether naturalization success of cultivated alien plants in Southern Africa is associated with climatic suitability and other plant characteristics, with a specific focus on the mediating role of climatic suitability. Naturalization success, defined as the incidence of naturalization among the species that have already been introduced in Southern Africa according to GloNAF (van Kleunen et al. 2019), was used as the response variable. The predictor variables included: geographical origin (dummy variable for native status in each of the TDWG-1 continents), native range size, plant traits (i.e., specific leaf area, height and seed mass), growth form (dummy variables for each category), phylogenetic distance to the native flora (PD_mean_ and PD_min_) and climatic suitability. All continuous variables were scaled to a mean of 0 and a standard deviation of 1 to enhance comparability among the estimates.

To thoroughly investigate these relationships and disentangle the indirect role of climate suitability, we used a two-step approach: we first employed a series of generalized linear models (GLMs) with binomial error distribution and a logit link function to assess the direct univariate and multivariate associations between naturalization success and all plant characteristics. This initial step provided a foundational understanding of the independent and joint associations between plant characteristics, climatic suitability, and naturalization success. Second, we conducted a causal mediation analysis to test whether climatic suitability mediates the relationship between significant plant characteristics and naturalization success (Baron and Kenny 1986; Kinlock et al. 2022; Pearl 2010; Ross et al. 2022). This analysis is essential for a comprehensive understanding of biological invasion mechanisms, as it allows us to quantify the direct and indirect effects of plant characteristics on naturalization success.

We proceeded with the mediation analysis for plant characteristics that showed a significant association with naturalization success. Because we only wanted to test the mediation effect on characteristics that have an association with climate suitability, we first modeled the associations between climatic suitability (i.e., number of predicted suitable grid cells in Southern Africa) and plant characteristics using generalized linear models with negative binomial error distribution and a logit link function. For the plant characteristics that show significant association with both naturalization success and climate suitability, we conducted a causal mediation analysis using the “MAZE” R package version 0.0.2 (Jiang et al. 2023). Plant characteristics were treated as independent variables (X), climatic suitability as the mediator (M), and naturalization success as the dependent variable (Y). This mediation analysis uses the fitted models for the dependent variable (i.e., naturalization success regressed on plant characteristics and climatic suitability) and mediator (climatic suitability regressed on plant characteristics). From these models, two effects are estimated: the indirect effects as the change in the estimates of the association between the independent and dependent variables when the mediator value changes (i.e., the effect of X on Y via M, keeping X at × 1 while M changes from M(× 0) to M(× 1)). 2) the direct effect as the change in the estimates of the association between the independents and dependent variables when the mediator value is fixed (effect of X on Y, keeping M fixed at M(× 0​) (Jiang et al. 2023). For continuous exposure variables, effects were evaluated by comparing the 10th percentile (× 0) to the 90th percentile (× 1), while for dummy variables, effects were compared between the presence (1) and the absence (0).

All analyses were done in R, version 3.6.1 (R Core Team 2019).

## Results

Of the 1,407 cultivated alien plants of Southern Africa used in this analysis, 492 (35%) had naturalized in at least one of the 18 subregions of Southern Africa.

### Naturalization success associations with plant characteristics

**Fig. 1:**
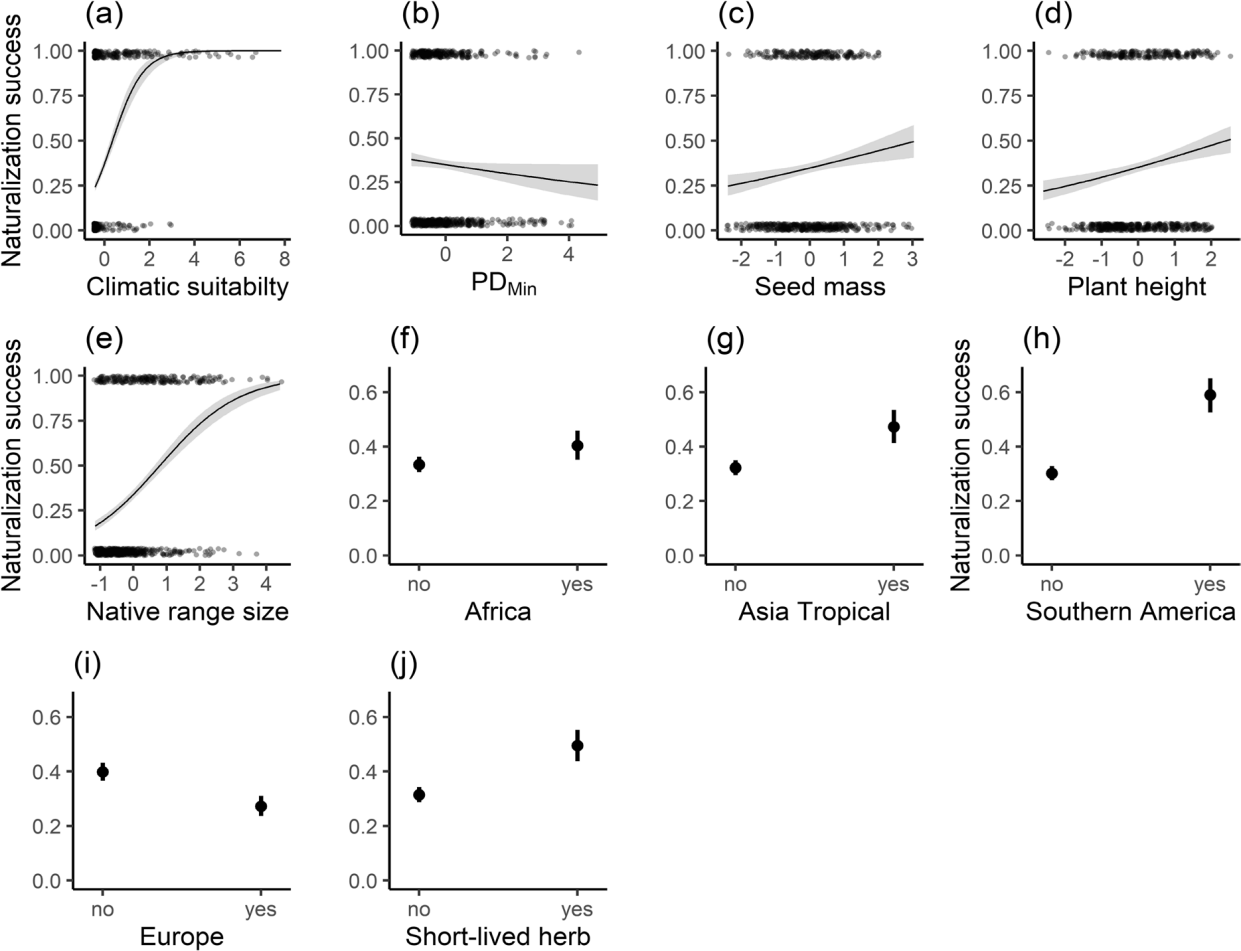
Naturalization success of cultivated alien plants in Southern Africa in relation to (**a**) climatic suitability, **b** phylogenetic distance to the nearest native relative (PDmin), **c** seed mass, **d** plant height, **e** native range size, **f** native status in Africa (**g**) native status in Tropical Asia, **h** native status in Southern America (**i**) native status in Europe and (**j**) having a short-lived herbaceous growth form. The solid lines and dots are the predicted means from binomial GLMs (Table S1), and grey shades and vertical lines indicate the 95% CIs. The values of continuous plant characteristics of naturalized and non-naturalized species are shown as points at one and zero, respectively (jittered to improve visibility). Statistics according to GLM models (two-sided tests) are indicated in the figure

Cultivated alien plants projected to have higher climatic suitability were more likely to naturalize in southern Africa (Fig. 1; Table S2). Among plant characteristics, without accounting for other variables, naturalization success also showed significantly positive association with native range size (ES = 0.84, *Z* = 12.56, *P* < 0.001), plant height (ES = 0.25, *Z* = 4.43, *P* < 0.001), seed mass (ES = 0.20, *Z* = 3.37, *P* < 0.001), short-lived herbaceous plants (ES = 0.76, *Z* = 5.61, *P* < 0.001), and species introduced from other parts of Africa (ES = 0.30, *Z* = 2.33, *P* = 0.020), Tropical Asia (ES = 0.64, *Z* = 4.55, *P* < 0.001) and Southern America (ES = 1.20, *Z* = 8.16, *P* < 0.001). Conversely, naturalization success showed a significant negative association with phylogenetic distance to the nearest native relative (PD_min_; Coefficient =  − 0.12, *Z* =  − 1.99, *P* = 0.047), and species native to Europe (ES =  − 0.57, *Z* =  − 4.82, *P* < 0.001).

### Multivariate associations between naturalization success and plant characteristics

**Fig. 2:**
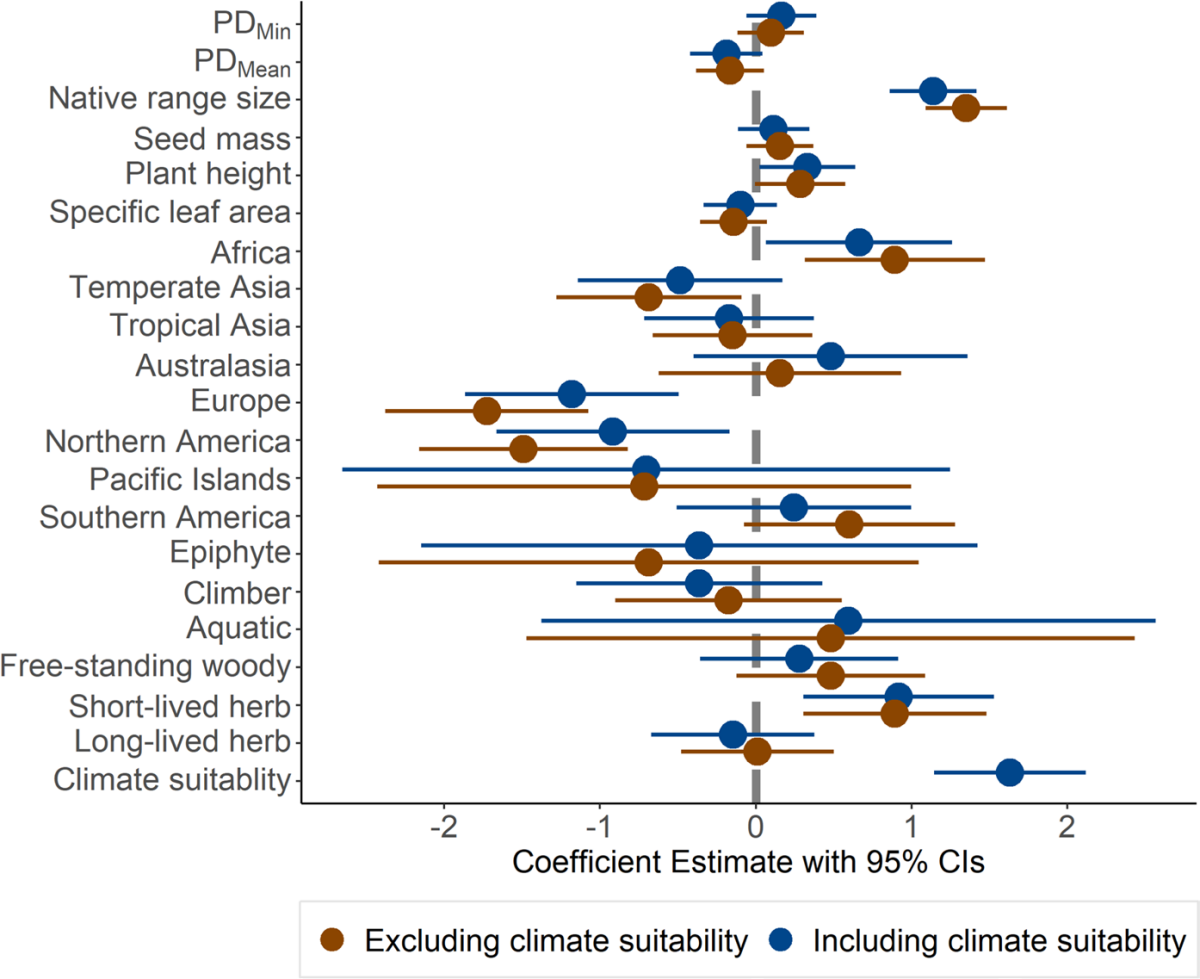
Partial regression coefficients estimated in generalized linear models (GLMs) of naturalization success in relation to the plant characteristics and climate suitability. Dots represent means and error bars are 95% confidence intervals (CIs). Blue and brown colors indicate models fitted including and excluding climate suitability, respectively

The full model with all plant characteristics, including climate suitability, has higher explanatory power (Nagelkerke’s pseudo *R*^2^ = 0.86, AIC = 702; Table S3) than the full model excluding climate suitability (*R*^2^ = 0.83, AIC = 773; Table S4). In the model where it was included, climate suitability again showed a strong positive association with naturalization success (ES = 1.63, *Z* = 6.56, *P* < 0.001). Naturalization success in the full model consistently showed significantly positive associations with native range size, plant height, short-lived herbaceous plants, and species introduced from other parts of Africa and a negative association with plants native to Europe. Most importantly, estimated coefficients of all plant characteristics showed a reduction in magnitude when climatic suitability was included in the model (Fig. 2; Table S3 and S4).

#### Climatic-suitability-mediated associations

**Fig. 3:**
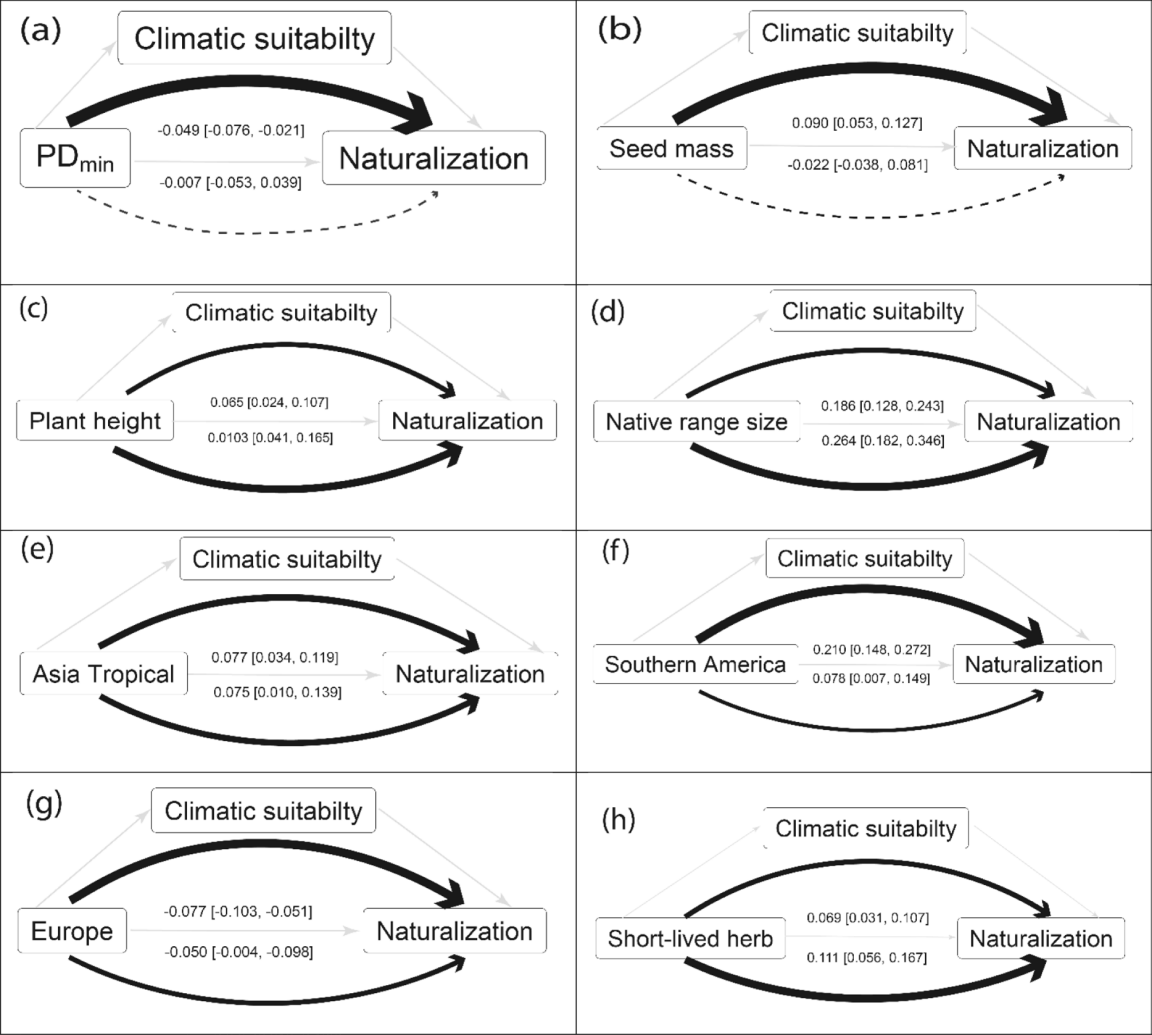
Direct and indirect relations between plant characteristics and naturalization success with climatic suitability as mediator. Theoretical paths between the response, predictors and mediator are shown in light grey arrows. Curved arrows show average indirect (upper curve), and direct (lower curve) effect estimated by comparing the 10th and the 90th percentiles of the contentious predictor in (**a**–**d**) and by comparing to the baseline values for the categorical values in (**e**–**h**). Dashed curved arrows indicated nonsignificant effects. The thickness of each curved arrow is proportional to its contribution to the overall predictor-response relationship

Our mediation analysis (Table 1, Fig. 3) revealed that climatic suitability has significant effect on the relationship between plant characteristics and naturalization success in Southern Africa. With the exception of species native to other parts of Africa, all significant associations between plant characteristics and naturalization success in Southern Africa were mediated through climatic suitability. The negative relationship between climatic suitability and phylogenetic distance to the nearest native relative and a native origin in Europe (Table S5) have indirectly mediated the negative association between these plant characteristics and naturalization success in Southern Africa (Table 1, Fig. 2a, g). In contrast, climatic suitability was positively associated with seed mass, plant height, native range size, native origins in Tropical Asia and southern America, and a short-lived herbaceous growth form (Table S5), and these associations indirectly mediated the positive associations between these plant characteristics and naturalization success in Southern Africa (Table 1, Fig. 2b–f, h). While the percentage of indirect mediating effect to total predictor-response relationship effect was the biggest (88%) in the association between naturalization success and phylogenetic distance to the nearest native relative, it was smallest (38%) in the association between naturalization and short-lived herbaceous growth form (Table 1).

**Table 1:** Results of the mediation analysis testing the direct and indirect effects of plant characteristics on naturalization success with climatic suitability as mediator. The average direct and indirect effects were estimated by comparing the 10th and the 90th percentiles of the contentious predictor to the baseline values for the categorical values. The 95% lower CI (CIL) and upper CI (CIU) were estimated from nonparametric bootstrapping. Bold font indicates significant effects

Response	Predictor	Mediator	Comparison	Indirect effect	CIL	CIU	Direct effect	CIL	CIU	Indirect/total proportion
Naturalization	PD_min_	Climatic suitability	− 0.90, 1.07	− 0.049	− 0.076	− 0.021	− 0.007	− 0.053	0.039	88%
	Seed mass		− 1.27, 1.23	0.090	0.053	0.127	0.022	− 0.038	0.081	80%
	Plant height		− 1.26, 1.52	0.065	0.024	0.107	0.103	0.041	0.165	39%
	Native range size		− 0.98, 1.43	0.186	0.128	0.243	0.264	0.182	0.346	41%
	Europe		Yes, NO	− 0.077	− 0.103	− 0.051	− 0.050	− 0.096	− 0.004	61%
	Tropical Asia		Yes, NO	0.077	0.034	0.119	0.075	0.010	0.139	51%
	Southern America		Yes, NO	0.210	0.148	0.272	0.078	0.007	0.149	73%
	Short-lived herb		Yes, NO	0.069	0.031	0.107	0.111	0.056	0.167	38%

## Discussion

We used a checklist of 1,407 alien plants introduced for cultivation in Southern Africa to test the direct effects of plant characteristics and geographical origins on naturalization success, as well as the indirect effects of the plant characteristics mediated by climatic suitability. As expected, our results showed that climatic suitability significantly increased naturalization success. Despite the moderately explained variation in naturalization success, we found substantial indirect effects of plant characteristics on naturalization success, mediated by climatic suitability. Our results collectively reveal the multifaceted interactions between climatic suitability, plant characteristics, and geographical origins in determining the naturalization success of cultivated alien plants in Southern Africa.

Our results support the widely accepted notion that climate matching between native and non-native ranges is fundamental for the naturalization success of alien plants (Darwin 1859; Elton 1958; Fristoe et al. 2023; Richardson and Pyšek 2012). We showed that climatic suitability was the most important predictor for naturalization success of cultivated alien plants in Southern Africa (*R*^2^ = 0.26; Table S2). Climatic suitability was significantly higher for naturalized compared to non-naturalized cultivated alien plants in Southern Africa. This also suggests that many naturalized species were introduced from climatically similar regions to Southern Africa. Our results also showed that species native to predominantly tropical regions such as South America, other parts of Africa and tropical Asia were more likely to naturalize compared to species introduced from temperate regions, like Europe. This relationship is again mediated by climatic suitability. In other words, species native to climatically similar regions to Southern Africa are more likely to naturalize because of their higher climatic suitability in Southern Africa. Moreover, our findings further support the feasibility of using climatic suitability analysis for invasion risk assessments (Dullinger et al. 2017; Feng et al. 2016; Haeuser et al. 2018; Mayer et al. 2017).

Consistent with previous studies, we found that the presence of phylogenetically closely related native species increased the naturalization success of cultivated alien plants in Southern Africa (Diez et al. 2009; Li et al. 2015; Omer et al. 2022; Procheş et al. 2008). However, this association shows very weak explanatory power (*R*^2^ = 0.004; Table S3), indicating the significant role of extrinsic factors affecting naturalization success. Moreover, the higher climatic suitability of cultivated alien plants with phylogenetically close native relatives, which we found in this study (Table S3), explains the positive relationship between phylogenetic relatedness to the native flora and naturalization success. This provides strong support for Darwin’s pre-adaptation hypothesis: an alien species is more likely to naturalize in regions with phylogenetically related native species, because the alien species is likely to possess traits similar to the ones that allow the related native species to occur in these regions (Darwin 1859).

The scientific literature consistently reveals a positive correlation between native range size and naturalization success (e.g., Fristoe et al. 2023; Omer et al. 2021; Prinzing et al. 2002). Here, we also found that species with larger native range sizes had higher probabilities of naturalization in Southern Africa. Species with large native ranges may possess greater ecological and genetic adaptability, which increases the naturalization probability of alien species once introduced to new ranges (Pyšek et al. 2009). This is further supported by the findings of our mediation analyses, that species with larger native range sizes also have higher climatic suitability in Southern Africa. However, native range size shows the biggest direct effect on naturalization success in our study. This might indicate that many other factors contribute to the naturalization success of species with large native range size. For example, species with large native range size are more likely to be prospected for cultivation and to be introduced more frequently (i.e., have a higher propagule pressure) (Omer et al. 2021).

Functional traits play an important role in defining the ecological strategies of plants, shaping how plant species respond to various environmental filters (Diaz et al. 2016; Oh et al. 2021). Our results showed that seed mass, plant height, and short-lived herbaceous species had significant positive associations with naturalization success. In a previous study, Omer et al. (2021) showed that species with intermediate seed mass and plant height are more likely to naturalize in Southern Africa. However, this nonlinear association was not significant in our study, which could be because of our smaller sample size or because of the different modeling approach. In the same study, consistent with our findings, short-lived herbs were also shown to have higher naturalization success in Southern Africa. However, this study showed that naturalization success was highest for cultivated plants with intermediate values of seed mass and plant height. This inconsistency might be because we only used a subset of the species used in Omer et al. (2021). However, in nutrient poor-habitats of Southern Africa, alien plants might take advantage of nutrient-rich large seeds and tall stature to survive in such harsh climatic conditions (Craine and Dybzinski 2013; Suárez-Vidal et al. 2017). This was further shown in our results, as species with larger seed mass and taller stature had higher climatic suitability in Southern Africa (Table S3). Possibly, short lived species are more likely to have relatively higher climatic suitability because they need only a short suitable season to complete their life-cycle (Sutherland 2004). However, these associations were again found to be partly mediated through climatic suitability, indicating the pivotal role of climate matching between native and non-native ranges for the naturalization of introduced plants.

Climatic similarity between native and non-native ranges has been widely recognized to enhance naturalization success (Fristoe et al. 2023; Richardson and Pyšek 2012). Nevertheless, climatic similarity alone does not entirely explain naturalization success, as species from other continents with climates similar to Southern Africa, like Australasia, were not more likely to be successful. The increased likelihood of naturalization for plants from specific continents might also be associated with other drivers, such as human cultivation preferences (Kinlock et al. 2022). Although all species in our analyses are cultivated in Southern Africa, some of them might be planted more frequently.

It is important to note that invasion ecology research has demonstrated variations in patterns and drivers of naturalization success across different spatial scales. (Park et al. 2020; Richardson and Pyšek 2012). Our study focused on the regional scale of Southern Africa, and thus, local-scale variations in alien species assemblages were not examined. The observed patterns and drivers may differ between organism groups and at different spatial scales. Therefore, our findings should be interpreted within the context of the regional scale of Southern Africa. Furthermore, our projection of climate suitability could also be associated with various sources of uncertainty, might affect the mediating role of climate in naturalization success. Additionally, our analysis lacks information on the introduction and cultivation history of species (e.g., residence time, propagule pressure), which are recognized as important drivers of naturalization (Kinlock et al. 2022). While we used the best available data, gaps in our functional traits data represent an additional source of uncertainty.

## Conclusions

Plant characteristics of the introduced cultivated floras in specific regions, such as Southern Africa, are not a random subset of the global species pool (Kinlock et al. 2022; Omer et al. 2021). These nonrandom subsets of cultivated species introduced from the global flora are further filtered by the environmental conditions in the new regions, as successfully naturalized species were found to have higher climatic suitability in Southern Africa compared to non-naturalized ones. Moreover, we showed that climatic suitability mediated almost all associations between plant characteristics and naturalization success in Southern Africa. Our study underlines the importance of considering the mediating role of climatic suitability in how plant characteristics drive the plant invasion process.

## Supplementary Information

Below is the link to the electronic supplementary material.

Supplementary file1 (DOCX 232 KB)

Supplementary file2 (CSV 195 KB)

Supplementary file3 (R 21 KB)

## Data Availability

Data used in this study is available as supporting information (Olivia et al. data).
