# Supplementary material for: Indirect role of climatic suitability in mediating the effects of plant characteristics on naturalization success of cultivated alien plants in Southern Africa
Source: Biol Invasions. 2025 Sep 25;27(10):220. doi: 10.1007/s10530-025-03677-3 (PMC12464098; doi:10.1007/s10530-025-03677-3)
Supplement: Supplementary file 1 — Supplementary file1 (DOCX 232 KB) [file 10530_2025_3677_MOESM1_ESM.docx]

**Indirect role of climatic suitability in mediating the effects of plant characteristics on naturalization success of cultivated alien plants in Southern Africa**

Sarah-Olivia Peter^1^, Franz Essl^1^, Bernd Lenzner^1^, Mark van Kleunen^2,3^, Ali Omer^1,4^

^1^Division of BioInvasions, Global Change & Macroecology, Department of Botany and Biodiversity Research, University of Vienna, Rennweg 14, Vienna 1030, Austria

^2^Ecology, Department of Biology, University of Konstanz, Universitätsstraße 10, D-78464 Konstanz, Germany

^3^Zhejiang Provincial Key Laboratory of Plant Evolutionary Ecology and Conservation, Taizhou University, Taizhou 318000, China

^4^Department of Forest Management, Faculty of Forestry, University of Khartoum; North Khartoum, 13314, Sudan

Correspondence:

Email: [ali.haroon.ali.omer@univie.ac.at](mailto:ali.haroon.ali.omer@univie.ac.at)

ORCID ID: <https://orcid.org/0000-0001-5687-3386>

**Supporting information** **S1: Climatic suitability assessment**

To assess the climatic suitability of Southern Africa for the cultivated flora, we used SDMs in the biomod2 platform, that is implemented in the ‘biomod2’ R package version 3.4-5 (Thuiller et al. 2020) to combine bioclimatic variables with presence records and randomly generated pseudo-absences.

We retrieved average global climate data for the time period from 1970 to 2000 in a 10' spatial resolution from WorldClim version 2.1 (Fick, Hijmans 2017). We selected the following five bioclimatic variables: (1) Temperature Seasonality, (2) Maximum Temperature of Warmest Month, (3) Precipitation of the Wettest Month, (4) Precipitation of the Driest Month, (5) Precipitation Seasonality. These variables were chosen based on their recognized influence on plant distributions (Root et al. 2003). In addition, we used human population density as a proxy of propagule pressure, which is available in the NASA Socioeconomic Data and Applications Center (Jones et al. 2020). These explanatory variables have pairwise Pearson’s r values below 0.70, and thus an acceptable effect of multicollinearity (Dormann et al. 2013).

Then, we used the species list of the cultivated flora of Southern Africa to collect data on global distributions (presence data) from the Global Biodiversity Information Facility (GBIF.org 2021; https://doi.org/10.15468/dl.9jsscb), using the ‘rgbif’ library in R (Chamberlain 2017). In order to not violate the assumption of niche conservatism and niche adaptation (Early, Sax 2014; Fernández, Hamilton 2015; Pearman et al. 2008), we considered native and alien occurrences. Presumably erroneous records (e.g. those that occur on ocean surfaces and in capitals), were automatically removed using the ‘CoordinateCleaner’ library in R (Zizka et al. 2019). Additionally, to avoid pseudoreplication, duplicated data points (multiple presence data points within the same 10′ × 10′ grid cells) were removed.

To define the potential current suitability of Southern Africa for the cultivated alien plants, we ran the SDMs using four modelling algorithms: two regression techniques, i.e., generalized linear models (GLM) and general additive models (GAM), and two classification techniques, i.e., random forest (RF) and boosted regression trees (BRT). We kept the default argument settings in the functions of these four modeling algorithms in biomod2. Since all presence only SDMs require presences and pseudo-absences (or background) data, we generated 10,000 random pseudo-absence records from all possible points within the global terrestrial surface. The random draw of pseudo-absence records was repeated three times, and equal weights were given in the models to presences and pseudo-absences. To evaluate our models, each model was separately ran three times using a split-sampling approach in which the data was split into 80% calibration and 20% evaluation datasets for each of the three pseudo-absence datasets (resulting in nine models per modelling algorithm and a total of 36 models for each species). We used the True Skill Statistic (TSS) to assess the predictive performance of the SDMs (Allouche et al. 2006). TSS ranges from −1 to 1, where 1 indicates perfect agreement, 0 indicates a random prediction and negative values indicate that predictions perform worse than random. The calibrated models have a very good performance with an average TSS value over 0.8.

Finally, we used the calibrated models to project the current climatic suitability in Southern Africa using a weighted mean ensemble forecast approach (Thuiller et al. 2009). We aggregated all the models of the repeated pseudo-absences and split-sampling into an ensemble projection to reduce uncertainties associated with each technique (Thuiller et al. 2009). The contribution of each model was weighted according to its TSS score (only models with TSS score > 0.5 were included). Then, the mean weighted ensemble was transformed into binary projections using a threshold that maximized the TSS to predict presences and absences. The resulting variable is therefore the sum of all suitable grid cells that each species is projected to encounter in Southern Africa.


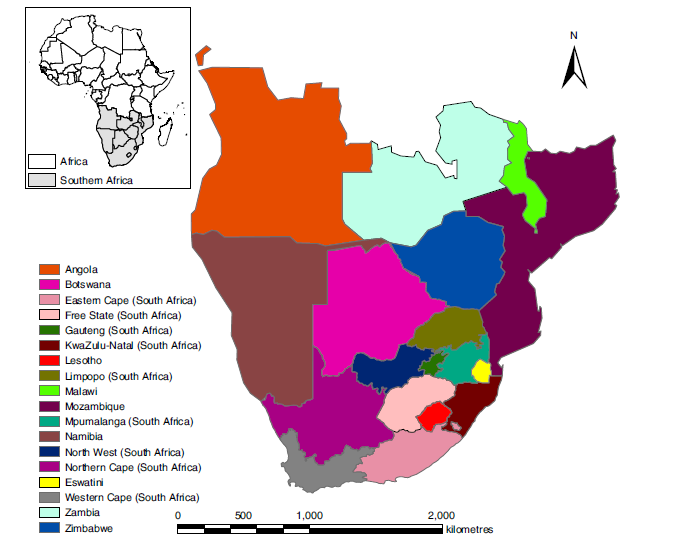


**Figure S1.** Map of Africa and the study regions showing the ten Southern African countries. Included in this map are Angola, Botswana, Eswatini (former Swaziland), Lesotho, Malawi, Mozambique, Namibia, South Africa, Zambia and Zimbabwe. The largest country, South Africa, is split into nine provinces; thus in total 18 different regions of Southern Africa were used (Omer et al. 2022).


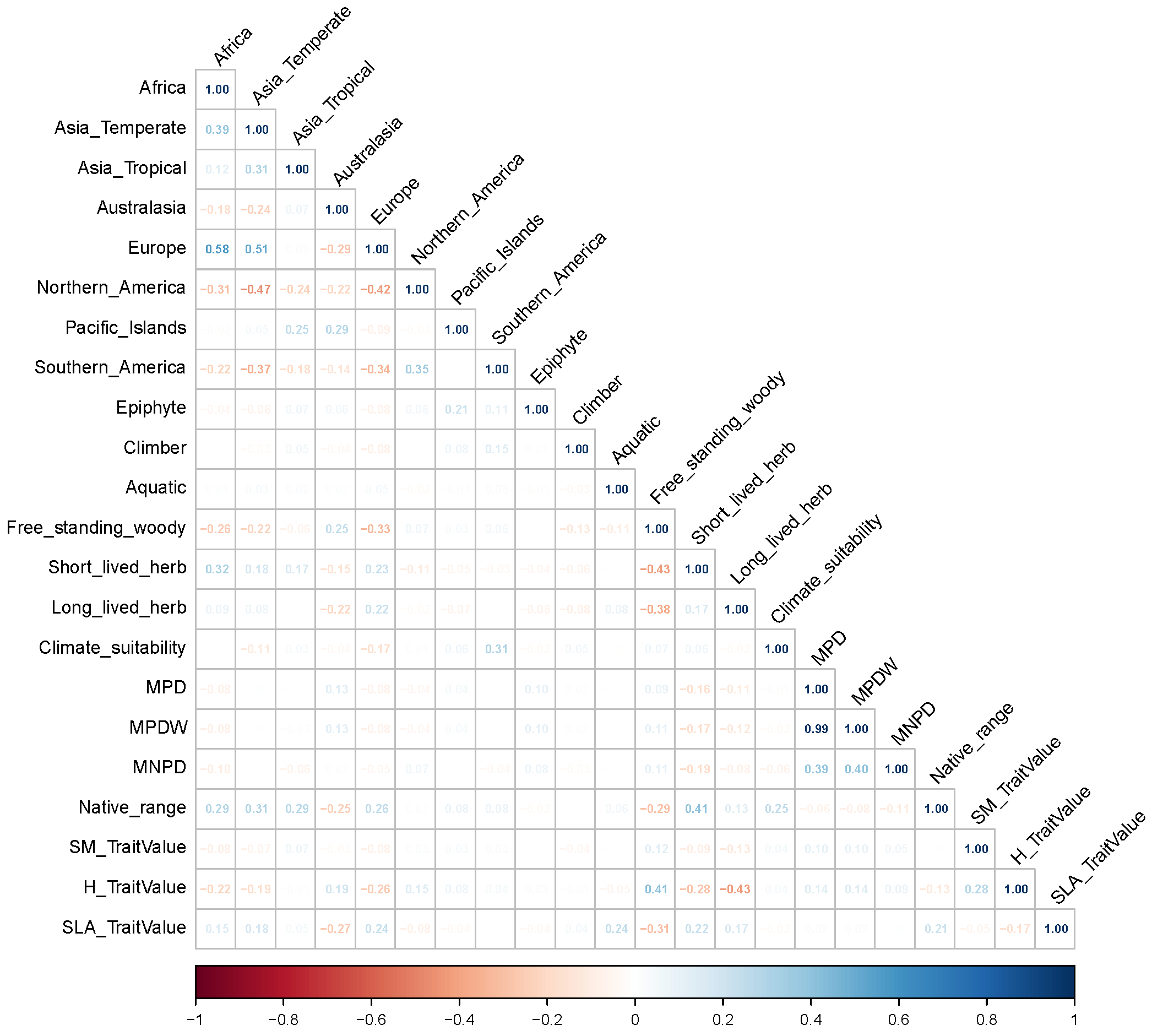


Figure S2: Correlation coefficients between the plant characteristics.

**Table S1.** Plant characteristics used as predictors of naturalization success. Description, sources and rational for inclusion.

| Plant characteristics | Description | Sources | Rational |
| --- | --- | --- | --- |
| Native origin | Whether a species is native to one of the 8 TDWG level 1 continents (the 9^th^ TDWG continent, Antarctica, was excluded as none of the species is native there). | Plants of the World Online database (POWO 2023; www.plantsoftheworldonline.org) | Species introduced from a region with environmental conditions similar to those in Southern Africa are more likely to naturalize. |
| Native range size | Numbers of TDWG level-3 regions (n = 369) to which each species is native. | Plants of the World Online database (POWO 2023; www.plantsoftheworldonline.org) | Species with large native ranges are more likely to have been introduced frequently, and they are likely to have a high environmental tolerance. |
| Phylogenetic distance | Phylogenetic distance between an introduced species and (1) the entire native flora, (2) the entire native flora weighted by the number of regions in which each native species occurs, and (3) the nearest native relative. | Constructed based on the phylogeny of seed plants (Smith, Brown 2018). | Phylogenetic relatedness to native flora should increase naturalization success, as it suggests that the alien species is likely to be preadapted. |
| Growth from | Whether a species belongs to one of the following growth-from categories: short-lived (i.e. annual or biennial), herb, long-lived herb, free-standing woody, aquatic, climber, epiphyte and parasite. | TRY database (Kattge et al. 2020) supplemented with other sources to close gaps in data coverage (see Omer et al. 2021) | Species with different growth form differ in stature, generation time and environmental requirements and that these factors may determine naturalization success. |
| Functional traits | seed mass, plant height and specific leaf area. | TRY database (Kattge et al. 2020) supplemented with other sources to close gaps in data coverage (see Omer et al. 2021) | These plant functional traits are indicative of characteristics e.g., seed dispersal, competitive ability, resource investment strategy chances of species naturalization that may determine naturalization success. |

**Table S2.** Results of the univariate binomial generalized linear models (GLMs) testing how the probability of naturalization success relates to climatic suitability and plant characteristics. Numerical predictor variables were standardized to a mean of zero and a standard deviation of one. Pseudo-R-squared (R^2^) values were calculated using Nagelkerke's method

| **Predictors** | **Estimate** | **SE** | **Z** | **P** | **R^2^** | **AIC** | **Predictors** | **Estimate** | **SE** | **Z** | **P** | **R^2^** | **AIC** |
| --- | --- | --- | --- | --- | --- | --- | --- | --- | --- | --- | --- | --- | --- |
| Intercept | -0.62 | 0.06 | -11.11 | <0.001 | 0.004 | 1821 | Intercept | -0.61 | 0.07 | -9.20 | <0.001 | 0.000 | 1825 |
| PD_min_ | -0.12 | 0.06 | -1.99 | 0.047 | 0.004 | 1821 | Northern America | -0.05 | 0.12 | -0.43 | 0.666 | 0.000 | 1825 |
| Intercept | -0.62 | 0.06 | -11.10 | <0.001 | 0.000 | 1825 | Intercept | -0.63 | 0.06 | -11.23 | <0.001 | 0.003 | 1822 |
| PD_mean_ | -0.03 | 0.06 | -0.48 | 0.631 | 0.000 | 1825 | Pacific Islands | 0.82 | 0.43 | 1.89 | 0.059 | 0.003 | 1822 |
| Intercept | -0.67 | 0.06 | -11.02 | <0.001 | 0.179 | 1630 | Intercept | -0.84 | 0.06 | -13.19 | <0.001 | 0.065 | 1757 |
| Native range size | 0.84 | 0.07 | 12.56 | <0.001 | 0.179 | 1630 | Southern America | 1.20 | 0.15 | 8.16 | <0.001 | 0.065 | 1757 |
| Intercept | -0.63 | 0.06 | -10.52 | <0.001 | 0.211 | 1605 | Intercept | -0.60 | 0.06 | -10.37 | <0.001 | 0.078 | 1745 |
| Seed mass | 0.20 | 0.06 | 3.37 | 0.001 | 0.211 | 1605 | Epiphyte | -0.44 | 0.48 | -0.93 | 0.353 | 0.078 | 1745 |
| Intercept | -0.61 | 0.06 | -10.65 | <0.001 | 0.076 | 1747 | Intercept | -0.64 | 0.06 | -10.60 | <0.001 | 0.080 | 1743 |
| Plant height | 0.25 | 0.06 | 4.43 | <0.001 | 0.076 | 1747 | Climber | 0.35 | 0.18 | 1.92 | 0.055 | 0.080 | 1743 |
| Intercept | -0.64 | 0.07 | -8.90 | <0.001 | 0.066 | 1091 | Intercept | -0.60 | 0.06 | -10.53 | <0.001 | 0.077 | 1746 |
| Specific leaf area | -0.06 | 0.07 | -0.89 | 0.374 | 0.066 | 1091 | Aquatic | 0.04 | 0.63 | 0.07 | 0.944 | 0.077 | 1746 |
| Intercept | -0.69 | 0.06 | -10.74 | 0.000 | 0.005 | 1820 | Intercept | -0.74 | 0.09 | -8.19 | <0.001 | 0.081 | 1742 |
| Africa | 0.30 | 0.13 | 2.33 | <0.001 | 0.005 | 1820 | Free-standing woody | 0.23 | 0.12 | 1.93 | 0.053 | 0.081 | 1742 |
| Intercept | -0.55 | 0.07 | -7.39 | <0.001 | 0.002 | 1823 | Intercept | -0.78 | 0.07 | -11.73 | <0.001 | 0.106 | 1715 |
| Temperate Asia | -0.16 | 0.11 | -1.42 | 0.157 | 0.002 | 1823 | Short-lived herb | 0.76 | 0.14 | 5.61 | <0.001 | 0.106 | 1715 |
| Intercept | -0.74 | 0.06 | -11.79 | <0.001 | 0.020 | 1805 | Intercept | -0.51 | 0.08 | -6.72 | <0.001 | 0.080 | 1743 |
| Tropical Asia | 0.64 | 0.14 | 4.55 | <0.001 | 0.020 | 1805 | Long-lived herb | -0.21 | 0.12 | -1.85 | 0.065 | 0.080 | 1743 |
| Intercept | -0.63 | 0.06 | -10.60 | <0.001 | 0.000 | 1825 | Intercept | -0.52 | 0.07 | -7.82 | <0.001 | 0.261 | 1530 |
| Australasia | 0.10 | 0.17 | 0.61 | 0.542 | 0.000 | 1825 | Climatic suitability | 1.47 | 0.12 | 12.03 | <0.001 | 0.261 | 1530 |
| Intercept | -0.41 | 0.07 | -5.92 | <0.001 | 0.023 | 1801 |  |  |  |  |  |  |  |
| Europe | -0.57 | 0.12 | -4.82 | <0.001 | 0.023 | 1801 |  |  |  |  |  |  |  |

**Table S3.** Results of the multivariate binomial generalized linear models (GLMs) testing how the probability of naturalization success relates to climatic suitability and plant characteristics. Numerical predictor variables were standardized to a mean of zero and a standard deviation of one. Pseudo-R-squared (R^2^) values were calculated using Nagelkerke's method.

| **Predictors** | **Estimate ± SE** | **Z** | **P** |
| --- | --- | --- | --- |
| Intercept | -0.21 ± 0.45 | -0.45 | 0.650 |
| PD_min_ | 0.16 ± 0.11 | 1.44 | 0.150 |
| PD_mean_ | -0.19 ± 0.12 | -1.59 | 0.110 |
| Climatic suitability | 1.63 ± 0.25 | 6.57 | <0.001 |
| Native range size | 1.14 ± 0.14 | 0.98 | <0.001 |
| Seed mass | 0.11 ± 0.12 | 2.11 | 0.330 |
| Plant height | 0.33 ± 0.16 | -0.82 | 0.040 |
| Specific leaf area | -0.1 ± 0.12 | 2.17 | 0.410 |
| Africa | 0.66 ± 0.31 | -1.45 | 0.030 |
| Temperate Asia | -0.49 ± 0.33 | -0.62 | 0.150 |
| Tropical Asia | -0.17 ± 0.28 | 1.07 | 0.540 |
| Australasia | 0.48 ± 0.45 | -3.38 | 0.280 |
| Europe | -1.18 ± 0.35 | -2.41 | <0.001 |
| Northern America | -0.92 ± 0.38 | -0.71 | 0.020 |
| Pacific Islands | -0.7 ± 1 | 0.63 | 0.480 |
| Southern America | 0.24 ± 0.38 | -0.40 | 0.530 |
| Epiphyte | -0.36 ± 0.91 | -0.90 | 0.690 |
| Climber | -0.36 ± 0.4 | 0.59 | 0.370 |
| Aquatic | 0.6 ± 1.01 | 0.86 | 0.550 |
| Free-standing woody | 0.28 ± 0.32 | 2.94 | 0.390 |
| Short-lived herb | 0.92 ± 0.31 | -0.55 | <0.001 |
| Long-lived herb | -0.15 ± 0.27 | 0.98 | 0.580 |
| AIC | 702 | R^2^ | 0.86 |

**Table S4.** Results of the multivariate binomial generalized linear models (GLMs) testing how the probability of naturalization success relates plant characteristics excluding climate suitability. Numerical predictor variables were standardized to a mean of zero and a standard deviation of one. Pseudo-R-squared (R^2^) values were calculated using Nagelkerke's method.

| **Predictors** | | **Estimate ± SE** | **Z** | **P** |
| --- | --- | --- | --- | --- |
| Intercept | | -0.31 ± 0.41 | -0.74 | 0.460 |
| PD_min_ | | 0.1 ± 0.11 | 0.89 | 0.370 |
| PD_mean_ | | -0.16 ± 0.11 | -1.49 | 0.140 |
| Native range size | | 1.35 ± 0.13 | 10.14 | 0.000 |
| Seed mass | | 0.15 ± 0.11 | 1.40 | 0.160 |
| Plant height | | 0.29 ± 0.15 | 1.94 | 0.050 |
| Specific leaf area | | -0.14 ± 0.11 | -1.30 | 0.190 |
| Africa | | 0.89 ± 0.3 | 3.03 | <0.001 |
| Temperate Asia | | -0.69 ± 0.3 | -2.26 | 0.020 |
| Tropical Asia | | -0.15 ± 0.26 | -0.57 | 0.570 |
| Australasia | | 0.16 ± 0.4 | 0.39 | 0.690 |
| Europe | | -1.73 ± 0.33 | -5.19 | <0.001 |
| Northern America | | -1.49 ± 0.34 | -4.36 | <0.001 |
| Pacific Islands | | -0.72 ± 0.87 | -0.82 | 0.410 |
| Southern America | | 0.6 ± 0.35 | 1.74 | 0.080 |
| Epiphyte | | -0.69 ± 0.88 | -0.78 | 0.440 |
| Climber | | -0.17 ± 0.37 | -0.47 | 0.640 |
| Aquatic | | 0.48 ± 1 | 0.48 | 0.630 |
| Free-standing woody | | 0.48 ± 0.31 | 1.56 | 0.120 |
| Short-lived herb | | 0.89 ± 0.3 | 2.98 | <0.001 |
| Long-lived herb | | 0.01 ± 0.25 | 0.04 | 0.970 |
| AIC | 773 | | R^2^ | 0.83 |

**Table S5.** Results of generalized linear models (GLMs) testing association between climatic suitability and plant characteristics. Numerical predictor variables were standardized to a mean of zero and a standard deviation of one.

| **Predictors** | **Estimate** | **SE** | **Z** | **P** | **Predictors** | **Estimate** | **SE** | **Z** | **P** |
| --- | --- | --- | --- | --- | --- | --- | --- | --- | --- |
| Intercept | 0.05 | 0.07 | 0.74 | 0.462 | Intercept | -0.12 | 0.08 | -1.51 | 0.132 |
| PD_min_ | 0.24 | 0.06 | 4.00 | <0.001 | Europe | 0.46 | 0.12 | 3.87 | <0.001 |
| Intercept | 0.04 | 0.06 | 0.69 | 0.491 | Intercept | -0.07 | 0.08 | -0.96 | 0.335 |
| PD_mean_ | 0.05 | 0.06 | 0.80 | 0.423 | Northern America | 0.43 | 0.13 | 3.32 | 0.001 |
| Intercept | -0.02 | 0.07 | -0.22 | 0.824 | Intercept | 0.06 | 0.07 | 0.98 | 0.327 |
| Native range size | -1.09 | 0.10 | -11.45 | <0.001 | Pacific Islands | -1.36 | 0.61 | -2.22 | 0.027 |
| Intercept | 0.08 | 0.07 | 1.10 | 0.272 | Intercept | 0.29 | 0.07 | 4.28 | <0.001 |
| Seed mass | -0.27 | 0.06 | -4.12 | <0.001 | Southern America | -1.49 | 0.19 | -7.91 | <0.001 |
| Intercept | 0.02 | 0.07 | 0.27 | 0.783 | Intercept | 0.04 | 0.07 | 0.52 | 0.600 |
| Plant height | -0.13 | 0.06 | -2.09 | 0.037 | Epiphyte | 0.24 | 0.46 | 0.52 | 0.601 |
| Intercept | 0.05 | 0.11 | 0.52 | 0.601 | Intercept | 0.10 | 0.07 | 1.52 | 0.129 |
| Specific leaf area | -0.13 | 0.08 | -1.58 | 0.114 | Climber | -0.64 | 0.21 | -3.08 | 0.002 |
| Intercept | 0.06 | 0.07 | 0.80 | 0.426 | Intercept | 0.04 | 0.07 | 0.59 | 0.558 |
| Africa | -0.05 | 0.14 | -0.40 | 0.689 | Aquatic | -0.02 | 0.66 | -0.04 | 0.972 |
| Intercept | 0.15 | 0.08 | 1.89 | 0.059 | Intercept | 0.06 | 0.10 | 0.61 | 0.541 |
| Temperate Asia | -0.21 | 0.12 | -1.79 | 0.074 | Free-standing woody | -0.03 | 0.12 | -0.24 | 0.809 |
| Intercept | 0.28 | 0.07 | 4.05 | <0.001 | Intercept | 0.19 | 0.07 | 2.65 | 0.008 |
| Tropical Asia | -1.34 | 0.18 | -7.33 | <0.001 | Short-lived herb | -0.72 | 0.15 | -4.74 | <0.001 |
| Intercept | -0.03 | 0.07 | -0.50 | 0.617 | Intercept | 0.07 | 0.08 | 0.84 | 0.403 |
| Australasia | 0.68 | 0.18 | 3.70 | <0.001 | Long-lived herb | -0.07 | 0.12 | -0.59 | 0.558 |

**References**

Allouche O, Tsoar A, Kadmon R (2006) Assessing the accuracy of species distribution models: prevalence, kappa and the true skill statistic (TSS). Journal of Applied Ecology 43:1223-1232

Dormann CF, Elith J, Bacher S, et al. (2013) Collinearity: a review of methods to deal with it and a simulation study evaluating their performance. Ecography 36:27-46

Early R, Sax DF (2014) Climatic niche shifts between species' native and naturalized ranges raise concern for ecological forecasts during invasions and climate change. Global Ecology and Biogeography 23:1356-1365

Fernández M, Hamilton H (2015) Ecological niche transferability using invasive species as a case study. PLOS ONE 10:e0119891

Fick SE, Hijmans RJ (2017) WorldClim 2: new 1-km spatial resolution climate surfaces for global land areas. International Journal of Climatology 37:4302-4315

GBIF.org (2021) GBIF Occurrence Download <https://doi.org/10.15468/dl.9jsscb>.

Jones B, O'Neill BC, Gao J (2020) Global 1-km Downscaled Population Base Year and Projection Grids Based on the Shared Socioeconomic Pathways. NASA Socioeconomic Data and Applications Center (SEDAC), Palisades, NY https://doi.org:https://doi.org/10.7927/Q7Z9-9R69

Omer A, Fristoe T, Yang Q, et al. (2022) The role of phylogenetic relatedness on alien plant success depends on the stage of invasion. Nature Plants 8:906-914

Pearman PB, Guisan A, Broennimann O, et al. (2008) Niche dynamics in space and time. Trends in Ecology & Evolution 23:149-158

Root TL, Price JT, Hall KR, et al. (2003) Fingerprints of global warming on wild animals and plants. Nature 421:57-60

Smith SA, Brown JW (2018) Constructing a broadly inclusive seed plant phylogeny. Am J Bot 105:302-314

Thuiller W, Georges D, Engler R, et al. (2020) biomod2: Ensemble Platform for Species Distribution Modeling. 3.4.6. https://CRAN.R-project.org/package=biomod2,

Thuiller W, Lafourcade B, Engler R, et al. (2009) BIOMOD – a platform for ensemble forecasting of species distributions. Ecography 32:369-373

Zizka A, Silvestro D, Andermann T, et al. (2019) CoordinateCleaner: Standardized cleaning of occurrence records from biological collection databases. Methods in Ecology and Evolution 10:744-751
